# Genetic Code Expansion and Bio-Orthogonal Labeling Reveal Intact HIV-1 Capsids inside the Nucleus

**DOI:** 10.1128/mbio.02346-22

**Published:** 2022-09-13

**Authors:** Leo C. James

**Affiliations:** a MRC Laboratory of Molecular Biology, Protein and Nucleic Acid Division, Cambridge, United Kingdom

**Keywords:** capsid, HIV-1, genetic code expansion, noncognate amino acid

## Abstract

Microscopy is one of the few techniques that can directly observe the HIV-1 capsid as it traverses the cell. However, an extrinsic or intrinsic label is needed to facilitate detection and this can perturb capsid behavior. Now, S. Schifferdecker, V. Zila, T. G. Muller, V. Sakin, et al. (mBio:e0195922, 2022, https://journals.asm.org/doi/10.1128/mbio.01959-22) have developed an ingenious direct labeling technology that uses genetic code expansion and click chemistry to produce infectious viruses whose capsids are labeled with only a single modified amino acid. Using this new system, together with electron tomography, the authors demonstrate that the capsid remains intact during its transport into the nucleus of T cells, supporting a late model of uncoating immediately before integration. Combining direct-labeled capsids with fluorescent nonstructural viral proteins or host cofactors promises to be hugely enabling for future studies. Moreover, the potential to install a bio-orthogonal label site specifically in the capsid is likely to have exciting applications beyond imaging.

## COMMENTARY

A key question in HIV-1 biology is when and where the virus puts together and takes apart its capsid. Most molecular biology methods can only assess this indirectly, with microscopy one of the few techniques with the potential to observe what the capsid looks like at different stages of its journey through the cell. However, microscopy is particularly sensitive to the observer effect: the perturbation of a system by its observation. This is because observing capsids inside the cell requires an extrinsic or intrinsic label to facilitate detection. Extrinsic labels include fluorescent antibodies (1) or fluorescent capsid binding proteins (e.g., CypA-DsRed [2]). These have provided enormous insight but can compete with endogenous cofactors, meaning they may prevent, or be prevented from, binding to capsid surfaces. This can cause observer effects such as the biased detection of those capsids not utilizing preferred cofactor-dependent transport pathways or the non-detection of those that are. Intrinsic labels are those introduced into the viral proteins themselves (3) and theoretically allow virions to be tagged without occupying cofactor-binding sites. For instance, an intrinsic label can be added to capsid through the addition of a dye-binding epitope (4) or by appending a fluorescent fusion protein (e.g., GFP [5, 6]). Unfortunately, these intrinsic labels can also be perturbing (7), as capsid is a complex higher-order structure whose requirement for self-assembly and disassembly enforces strict stereochemical tolerances. Intrinsic labeling strategies therefore usually rely on making heterogeneous chimeric capsids, with structural and functional consequences that can be hard to define.

**FIG 1:**
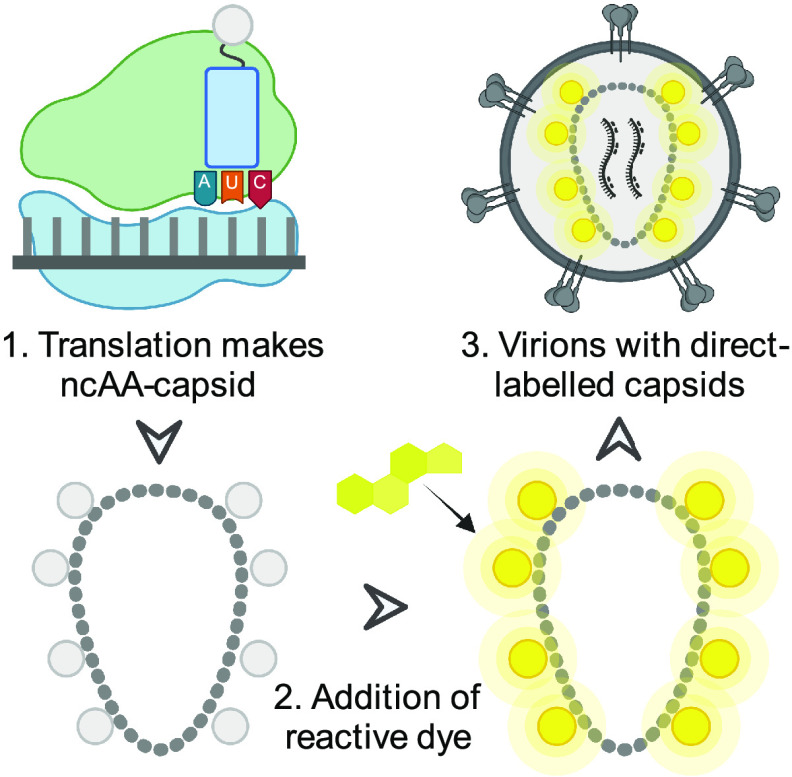
Genetic code expansion allows production of infectious HIV-1 with a prelabeled capsid. (1) A modified Gag gene with an amber stop is decoded by the ribosome to install a noncognate amino acid (ncAA). (2) A cell-permeable dye is added which reacts with the ncAA-Gag inside virus-producing cells. (3) Fluorescent labeled Gag is packaged into newly forming virions and assembles into a fluorescent capsid upon budding.

In their recent article in *mBio*, Schifferdecker and colleagues (8) present an ingenious direct labeling system that uses genetic code expansion and click chemistry to produce infectious viruses whose capsids are labeled with a single modified amino acid. The authors accomplished this by introducing an amber stop codon into position 14 in the capsid and then producing virions in cells expressing a modified tRNA and cognate synthetase to incorporate a cyclopropene lysine rather than a stop at the amber codon (Fig. 1). Homogeneity of incorporation is ensured because only CA containing the noncognate amino acid (ncAA) is produced as a full-length protein. Using this system, the authors produced a ncAA-virus that assembles and buds from cells comparably with wild type.

Installation of a single cyclopropene lysine within CA provides a unique dienophile chemical handle that can be reacted with tetrazine-containing compounds in efficient and biocompatible inverse electron demand Diels-Alder reactions (9). In their study, Schifferdecker et al. (8) added the membrane-permeable dye silicon rhodamine tetrazine (SiR-Tet) to viral supernatants to produce capsids with a fluorophore that has ideal properties for superresolution microscopy. Fluorescence intensity measurements confirmed that this results in >95% of labeled viral particles. More remarkably still, the infectivity of labeled virus was only reduced by 50% compared to unlabeled wild type.

Schifferdecker et al. benchmarked their new labeling system against standard immunofluorescence microscopy in a series of experiments. Comparing labeled virus with unlabeled wild type revealed no difference in transport kinetics to the nucleus, with similar numbers of particles detected at the nuclear envelope, although there were fewer labeled capsids within the nucleus itself suggesting reduced nuclear import efficiency. These differences notwithstanding, the fact that labeled particles could be detected within the nucleus allowed the authors to use stimulated emission depletion (STED) microscopy to determine whether capsids in the nucleus are intact. At an MOI of 0.8, individual particles rather than clusters could be resolved, and measuring their fluorescence intensity demonstrated that capsids have the expected quantity of CA for an intact structure. This is a critical finding and provides important evidence that the HIV-1 capsid only uncoats after nuclear import. Commendably, the authors extended their studies into primary human CD4^+^ T cells. Clever use of the capsid binding small molecule PF74 allowed them to confirm that binding of the cofactor CPSF6 prevents the immunofluorescence detection of CA particles inside the nucleus. This is a further important result as it highlights that a failure to detect intact capsids in the nucleus can be due to epitope masking rather than the inability of capsids to pass intact through the nuclear pore. Indeed, the authors provide some of the most compelling data to date that capsids enter the nucleus of T cells intact: fluorescence intensity measurements consistent with a full complement of CA proteins and correlative electron tomography confirming a complete conical morphology. Together with previous morphology studies (10) and live microscopy using a fluid-phase marker (11) (in which a fluorescent protein is trapped within the capsid and released only when a sufficiently large hole in the capsid is made), the evidence that the HIV-1 capsid remains as intact as it is possible to measure until immediately before integration is becoming hard to dispute.

Microscopy continues to provide crucial insight into HIV-1 infection and the direct-labeling technology developed by the Müller lab is a key advance that will be hugely enabling for future studies. In addition to reducing the observer effect, direct labeling may help in differentiating infectious from defective particles inside the cell, a common criticism of microscopy data. Various approaches have been taken to tackle this problem, such as utilizing low multiplicities of infection (12) or tracking individual particles in live cells until immediately before integration (6). Combining direct-labeled capsids with viruses containing other labeled proteins (e.g., integrase [13]) or fluorescent host cofactors (11) is likely to offer further insight into the precise timing of uncoating. The ability to quantify the amount of CA protein associated with each capsid also provides a powerful complement to morphological characterization (10), particularly when studying controversial steps such as progress through the nuclear pore. Looking beyond the immediate question of where and when capsid uncoats, the present study also illustrates the feasibility and power of genetic code expansion in studying HIV-1 infection. The potential to install a bio-orthogonal label site specifically anywhere in the capsid is likely to have many further applications beyond imaging.
